# Structure and Mechanical Properties of Cu–Al–Mn Alloys Fabricated by Electron Beam Additive Manufacturing

**DOI:** 10.3390/ma16010123

**Published:** 2022-12-22

**Authors:** Evgeny Moskvichev, Nikolay Shamarin, Alexey Smolin

**Affiliations:** Institute of Strength Physics and Materials Science, Siberian Branch Russian Academy of Sciences, 2/4, pr. Akademicheskii, Tomsk 634055, Russia

**Keywords:** shape memory alloy, electron beam additive manufacturing, heat input, microstructure, mechanical properties, tribological performance

## Abstract

In this work, the method of electron beam additive manufacturing (EBAM) was used to fabricate a Cu-based alloy possessing a shape memory effect. Electron beam additive technology is especially relevant for copper and its alloys since the process is carried out in a vacuum, which makes it possible to circumvent oxidation. The main purpose of the study was to establish the influence of the printing parameters on the structure of the obtained products, their phase composition, mechanical properties, dry friction behavior, and the structure-phase gradient that formed in Cu–Al–Mn alloy samples during electron beam layer-by-layer printing. The results of the study allowed us to reveal that the structure-phase composition, the mechanical properties, and the tribological performance of the fabricated material are mainly affected by the magnitude of heat input during electron beam additive printing of Cu–Al–Mn alloy. High heat input values led to the formation of the β1′ + α decomposed structure. Low heat input values enabled the suppression of decomposition and the formation of an ordered 1 structure. The microhardness values were distributed on a gradient from 2.0 to 2.75 GPa. Fabricated samples demonstrated different behaviors in friction and wear depending on their composition and structure, with the value of the friction coefficient lying in the range between 0.1 and 0.175.

## 1. Introduction

Copper has good ductility, electrical conductivity, and thermal conductivity, which allows it to be used in a wide range of industries. In particular, copper-based alloys are traditionally used as materials for tribo-conjugations and friction units. Among all copper alloys, it is worth noting such an important class of materials as the shape memory alloys (SMA), the interest in which has recently increased [1,2,3,4,5,6,7].

In Cu–Al alloys, the β phase of the bcc A2 structure is formed directly from the melt. Furthermore, it undergoes an order–disorder transition: A2 (disordered bcc Cu) → B2 (CuAl) → D0_3_ (Cu_3_Al) [8]. Phase transformation-induced effects of shape memory and superelasticity consist of phase transitions from the high-temperature austenite phase to the low-temperature martensite phase and vice versa [9]. This is the main characteristic responsible for the properties of this class of alloys [10]. Phase transition temperatures for aluminum bronze are strictly dependent on the chemical composition of the alloy, as shown elsewhere [4,11,12,13].

Cu–Al–Mn alloys differ favorably from other copper-based SMA due to alloying with manganese. Manganese increases mechanical and corrosion properties and improves the technological characteristics of Cu–Al alloys. It increases not only strength, but also plasticity and, consequently, the ability for pressure-assisted machining. Bronze Cu–9Al–2Mn is well treated by pressure in both hot and cold conditions, whereas bronzes Cu–9Al–3Fe, Cu–10Al–3Fe–1Mn, and Cu–10Al–4Fe–4Ni are well deformed only in the hot state. At the same time, manganese significantly affects the formation of the beta phase, expanding the temperature range of its existence [9]. In tribology, martensitic transformations are considered new mechanisms of friction and structural adaptability of the material to loading conditions [14,15,16]. Dependence of the mechanical properties of these alloys on their structure was studied in [17,18]; to enhance these properties, it was suggested to use the addition of different elements.

Methods that are the most widely used for manufacturing Cu–Al–Mn bronze products include powder metallurgy and vacuum casting. At the same time, methods of local metallurgy and additive technologies are rarely represented in the literature in relation to such alloys, although they have wide possibilities for manufacturing materials with specific characteristics. Since these technologies allow varying the chemical composition of the material during layer-by-layer printing, they make it possible to create a structure-phase gradient with the required physical and mechanical properties in the finished product or semi-finished product directly in the local area exposed to load. This is especially topical for all Cu–Al alloys widely used in tribotechnical applications [19,20,21].

In this work, the method of electron beam additive manufacturing (EBAM) was used to fabricate wear resistant Cu–Al–Mn samples. Electron beam technology is particularly relevant for copper and its alloys since the process is carried out in a vacuum, which makes it possible to circumvent oxidation. The structure formation under the conditions of local fusing that characterize additive manufacturing is a complex process. However, additive manufacturing methods are now being increasingly used. As a result, understanding the structure formation in additive manufacturing technology has become a hot research topic. Due to the use of manganese aluminum bronze in tribo-conjugations, wear behavior analysis of fabricated samples also needs to be performed.

Because of their complex crystallization process, aluminum bronze alloys are very sensitive to technology and production parameters [9]. Recently, a large number of papers have appeared devoted to the additive manufacturing of nickel aluminum bronze [22,23], while much fewer studies have dealt with the additive manufacturing of manganese aluminum bronze.

The main purpose of the study was to establish the influence of the printing process parameters on the structure of the obtained products, their phase and chemical composition, mechanical properties, wear performance, and the structure-phase gradient that formed in Cu–Al–Mn alloy samples during electron beam layer-by-layer printing.

## 2. Materials and Methods

Commercial Cu–7Al aluminum bronze wire was selected for additive printing of the lower layers of the workpiece. Cu–Al bronze was chosen as the underlayer because it has a similar chemical composition, which excludes the formation of intermetallic compounds and the appearance of particles of a material other than aluminum bronze in the fusion zone of the layers of the underlayer material and Cu–Al–Mn layer. AISI 321 stainless steel was used as a substrate because it has a good ability to fuse with aluminum bronze. Substrate is a consumable material from which the finished product is cut off after printing is completed. Two Cu–Al–Mn alloys were used to print the upper layers; their chemical composition is given in Table 1. Cu–Al–Mn bronze was originally supplied in ingots manufactured by the standard metallurgical method. For additive printing, the ingots were cut into bars of the size of 3 × 3 × 20 mm, after which the surfaces of the bars were ground using sandpaper of 240 grit.

For printing, a laboratory EBAM machine manufactured at the Institute of Strength Physics and Materials Science of the Siberian Branch of the Russian Academy of Sciences was utilized. This machine has been successfully used for 3D printing of various metal and alloy products with interesting properties that have been thoroughly studied and discussed elsewhere [24,25]. The main unit of the machine is an electron gun with a thermionic cathode in the form of a tantalum tablet with indirect heating. The accelerating voltage of this gun is 30 kV. The electron gun is equipped with a system for focusing and moving the electron beam. It is also possible to form various scans of the electron beam (ellipse, spiral, line, etc.). The printed material is usually fed in the form of a wire.

The printing process on the EBAM machine consists of the following steps:

- A substrate is installed on a copper water-cooled table.
- The focus of the electron beam is adjusted to the surface of the substrate.
- The wire feed mechanism is adjusted so that the wire falls into the area of the electron beam on the substrate (the wire is fed at an angle of ~45° to the table surface in the center of the beam).
- A working pressure (vacuum) is created.
- Printing is performed according to the specified algorithm for the formation of layers.

When printing, the electron beam forms a melt bath on the surface of the substrate/sublayer where the wire is fed, and the table is moved along three coordinates in accordance with the chosen printing strategy.

The preferred type of material transfer is continuous transfer, when the wire melts upon contact with the melt bath or its proximity without forming droplets. Drop transfer is less preferable because, in this case, it is hard to control the geometric shape of the product, and drops of molten filament can be greatly overheated, which in turn can lead to evaporation of some elements (in the presence of low-melting elements, a drop can spray without getting into the melt bath). Moreover, in some cases, radiation from the electron beam impact zone can repel droplets of molten filament.

Printing is carried out in a vacuum with a working pressure of 5 mPa. To remove excess heat energy during operation, the workpiece is formed on a substrate (in this work it is steel) located on a copper table with liquid cooling.

**Figure 1:**
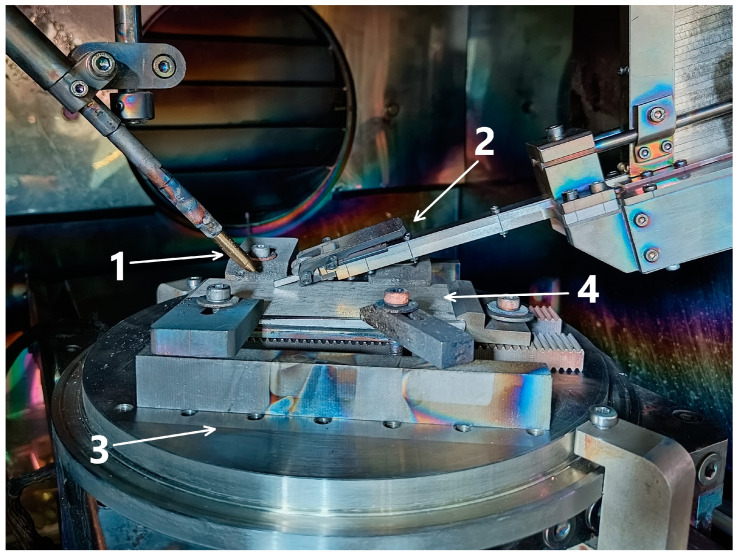
Configuration of the camera of the EBAM machine for printing by a combined method with wire and bar feeding of the material: (1) wire feeder, (2) bar feeder, (3) cooled table, (4) steel substrate.

The bar method of material feeding during additive printing of Cu–Al–Mn layers was used in this work. This method has a higher productivity in comparison with wire feed and allows us to work with a wide range of materials, not limited to wire filaments. However, Cu–7Al layers were printed using the standard wire feed method. The printing process was arranged as follows: a Cu–7Al layer of the required height was applied to the steel substrate by using wire feed method. The wire feeder was removed from the table, a bar feeder was supplied, and further Cu–Al–Mn printing was carried out using bars. The image of the EBAM machine camera at the start of the process is shown in Figure 1.

The combined printing method described above makes it possible to create layers of material with a structure-phase gradient by controlling the temperature regime of printing and the depth of the mixing zone, and it also provides ample opportunities for creating layered structures with alternating layers of different materials.

**Figure 2:**
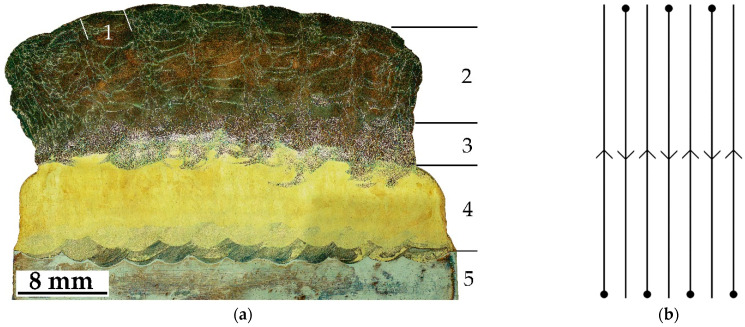
(**a**) Optical image of the bulk sample cross-section: (1) single track pool; (2) Cu–Al–Mn stable layers; (3) transition zone; (4) Cu–Al layers; (5) steel substrate. (**b**) Scheme of the printing strategy.

The macrostructure of the obtained samples is depicted in the optical image of the typical cross-section shown in Figure 2a. The sample is fabricated in accordance with the specified printing strategy; therefore, the macrostructure of the cross-section is represented by crystallized melt pools 1 with a width of 3–4 mm (clearly seen in Cu–Al–Mn layers). This is because, in this work, a printing strategy with intermittent application of parallel layers was used (Figure 2b); when between passes, the sample was kept for a short time (10 s corresponding to dots in Figure 2b), required for changing the bar in the feeder, changing the printing track, and cooling the sample. As a result, boundaries were formed between adjacent tracks consisting of smaller grains crystallized at a higher speed. It is also worth noting the transition zone 3 in Figure 2a, formed at the border of the fusion of two bronzes.

In the process of fabricating samples, the most optimal regime of printing and postprocessing of the workpiece was selected to obtain the required parameters of the structure and chemical composition.

The calculation of heat input *E* during printing was carried out according to the following formula:

(1) $$E=\frac{60\times U\times I}{1000\times V},$$

where *U* is the voltage (kV), *I* is the current (mA), and *V* is the print speed (mm/min).

The selected parameters of the printing regimes are shown in Table 2.

When printing samples, it was found that the optimal regimes for obtaining intact Cu–Al–Mn samples were regimes 1–3 since printing in the regime 0 with high heat input led to an increase in the edge effect of the workpiece and, accordingly, its irregular shape. Printing in the low heat input regime, with high print speed (regime 4), led to the absence of fusion of bronze layers, as well as the formation of discontinuities and pores between the layers. After fabrication, some samples were subjected to homogenization annealing at a temperature of 900 °C for 4 h, followed by cooling into water.

Thus, hereinafter, we denote the fabricated samples as *X*-*Y*AS, where *X* is the raw alloy (ingot) number in Table 1, and *Y* is the regime number in Table 2. The annealed samples are correspondingly denoted as *X*-*Y*AN.

A field-emission scanning electron microscope (FE SEM) Tescan MIRA 3 LMU (TESCAN ORSAY HOLDING, Brno, Czech Republic) equipped with Oxford Instruments Ultim Max 40 EDS detector (Oxford Instruments, High Wycombe, UK) was used to analyze the structure of the materials fabricated in this work. The structure analysis was carried out with the following parameters: accelerating voltage, 20 kV; beam current, 1.6 nA. The analysis results were processed in AZtec v. 4.2 licensed software (Oxford Instruments, High Wycombe, UK). The samples for SEM analysis were cut in a direction perpendicular to the printing plane and were prepared by diamond polishing until 1 µm followed by silica polishing. The same samples were used later to measure microhardness.

The crystal structure of the samples was studied by X-ray diffraction using Shimadzu XRD-7000S (Shimadzu, Kyoto, Japan) in a Bragg–Brentano configuration using Cu–Kα1 radiation at 40 kV and 30 mA. The phase composition of the samples was identified using PDF-4+ 2015 software (ICDD, Newtown Township, PA, USA). The samples for XRD studies with dimensions of 10 × 10 mm were cut out in the printing plane from the uppermost layer; their surface was ground mechanically using sandpaper (400–2000 grit) followed by diamond polishing to 6 µm.

Microhardness was measured using a “Duramin-5” (Struers A/S, Ballerup, Denmark) microhardness tester at 100 g load. Wear tests were evaluated on a pin-on-disc tribometer (Tribotechnic, Clichy, France) operated at 0.1 m/s speed with a 20 N load for 90 min. Discs made of 440C steel were used as counterbodies.

## 3. Results and Discussion

### 3.1. Microstructure of the Obtained Samples

**Figure 3:**
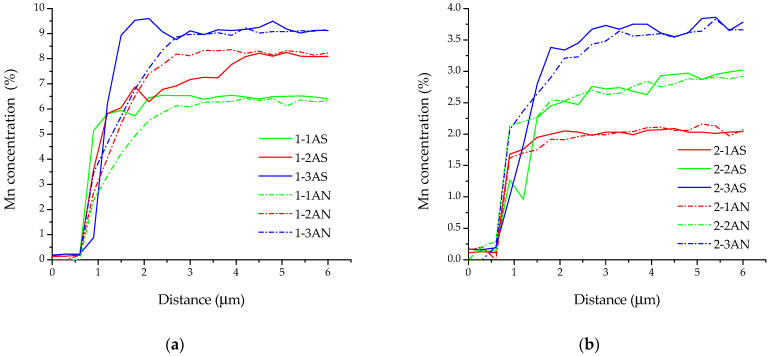
Concentration of manganese in the height of the samples in the state after printing (*X*-*Y*AS) and after annealing (*X*-*Y*AN): (**a**) fabricated from Alloy 1; (**b**) fabricated from Alloy 2.

A set of EDX spectra were analyzed from square sections with a side of 250 µm and a step of 250 µm, starting from the substrate and finishing at the upper part of the sample. During the analysis, it was found out that the structure of the samples had a directional gradient of chemical composition (Figure 3). The manganese content depended on the value of the heat input and determined the structure-phase gradient formed in the samples. It is also worth noting the difference in the width of the gradient zone from the Cu–7Al substrate to the Cu–Al–Mn layers. In general, the gradient area could be divided into the following characteristic zones: (i) a zone of a sharp increase in the content of Mn; (ii) a zone of instability; (iii) a zone with a stable content of alloying elements. Annealing after printing significantly affected the structure of the gradient zone, leveling the chemical composition in the transition zone from Cu–7Al to Cu–Al–Mn. On the basis of the EDX analysis data, it was revealed that the pronounced transition zone formed during the printing and characterized by an uneven phase composition, as well as a reduced Mn content, was leveled during annealing, as can be seen in the plots in Figure 3. Due to the temperature treatment, it was possible to homogenize this area; however, in the case of Cu–11Al–9Mn alloy, this led to a blurring of the chemical composition of the transition zone (Figure 3).

It should be noted that, when printing in regimes with high heat input (1, 2), manganese burned out, whereas printing in regime 3 did not lead to significant losses (Figure 3). This effect is a well-known problem of temperature treatment or fabrication of metal materials.

Table 3 shows the data of quantitative EDX analysis collected from the top layer of samples. Sections of 250 × 250 µm were analyzed to certify the obtained material. The data in Table 3 show that a homogeneous layer of material was obtained at a distance of 2–3 mm from the Cu–7Al substrate as a result of the formation of a transition zone in the samples. This transition zone, in turn, was caused by mixing the deposited layers and substrate Cu–7Al layers.

**Figure 4:**
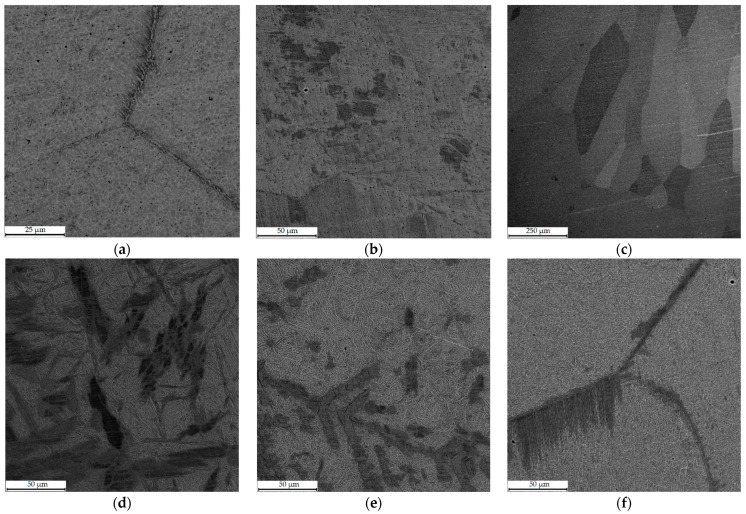
SEM-BSE images of as-printed samples microstructure: (**a**–**c**) for Alloy 1, 1–3 regimes, respectively; (**d**–**f**) for Alloy 2, 1–3 regimes, respectively.

**Figure 5:**
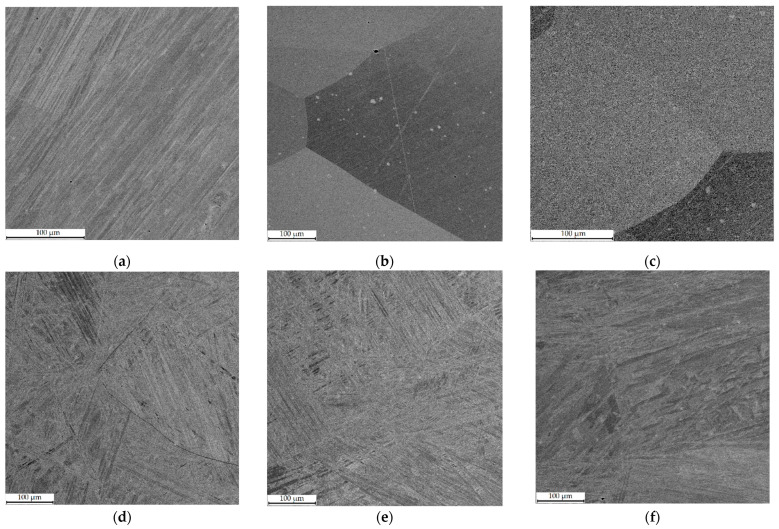
SEM-BSE images of annealed samples microstructure: (**a**–**c**) for Alloy 1, 1–3 regimes, respectively; (**d**–**f**) for Alloy 2, 1–3 regimes, respectively.

Figure 4 depicts SEM micrographs of Cu–Al–Mn alloy samples immediately after printing in different regimes, and Figure 5 shows the same samples after annealing. The structure of Cu–11Al–4Mn alloy samples represents the consequences of the eutectoid decay of the β-phase. The ratio β/α depends on the printing regime since light elements burn out during the printing process, which also affects the width of the β-phase formation zone [26,27]. In the printing regime with low heat input (sample 2–3 in Figure 5f), crystallization occurs quickly due to the cooling of the table and the high print speed; the decay does not have time to occur sufficiently. The structure is mainly represented by the martensitic phase, with the formation of the α-phase only along the boundaries. Printing regimes with medium and high heat input lead to a greater degree of manganese burnout, greater heating of the sample and, as a result, a greater proportion of the formed grains of the α-phase (Figure 5d,e).

Due to the initially higher amount of manganese in Cu–11Al–9Mn alloy samples, crystallization from the high-temperature β-phase occurred with the formation of an ordered β_1_-phase [26,27]. Printing in a low-heat-input regime (samples 1–3 in Figure 5c) with quick cooling allowed suppressing eutectoid decomposition and obtaining a sample with large grains of the ordered β_1_-phase (except the transition zone in which, due to mixing with Cu–7Al substrate, the alloy was depleted) [28,29]. Printing regimes with medium and high heat input led to the decomposition of the ordered β_1_-phase and the formation of the α + β_1_′ system, similar to samples from Alloy 2 (Cu–11Al–4Mn). At the same time, the grain structure of sample 1-2 (Figure 5b) was characterized by both the presence of large grains characteristic of the ordered β_1_-phase and a two-phase structure, as a consequence of the influence of the width of the transition zone.

The samples fabricated from Alloy 2 (Cu–11Al–4Mn) and subjected to annealing were characterized by a homogeneous needle-shaped martensitic structure with a well-defined interface between Cu–7Al layers and Cu–Al–Mn layers. After annealing, the samples fabricated from Alloy 1 (Cu–11Al–9Mn) had different structures depending on the printing regime. Thus, sample 1-1, printed with high heat input, transformed into martensite completely, sample 1-3, printed with low heat input, remained unchanged, and the intermediate sample 1-2, due to its wide transition zone, was divided into two parts, the lower of which (located closer to Cu–7Al) transformed into martensite, and the upper remained in the β_1_ phase.

### 3.2. Microhardness of the Obtained Samples

**Figure 6:**
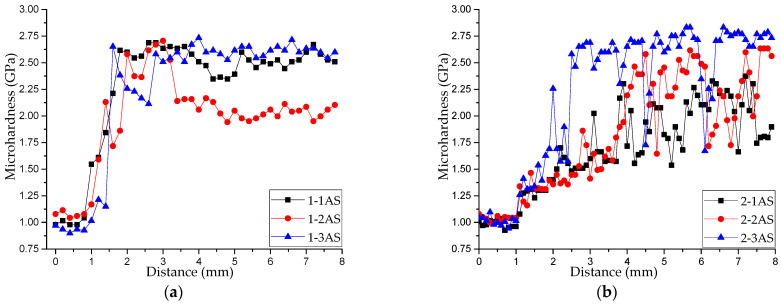
Distribution of the microhardness over the height of the samples printed from Alloy 1 (**a**) and Alloy 2 (**b**).

Figure 6 presents the distributions of the microhardness along the height in all printed samples. According to an analysis of the data from Figure 3 and Figure 6, the availability and width of the transition zone produced a structure-phase gradient that resulted in a gradient distribution of hardness in the samples. Samples printed from Alloy 1 (Cu–11Al–9Mn) were characterized by a specific zone of increased hardness (Figure 6a), a sharp increase in hardness in the transition zone, and its uniform distribution in the rest of the printed layer. Samples printed using bars from Alloy 2 (Cu–11Al–4Mn) were characterized by a smooth increase in hardness in the transition zone and an uneven distribution of hardness in the upper part of the samples (Figure 6b). This unevenness was due to the presence of a soft α-phase and a martensitic phase with high microhardness and excellent wear resistance [16]. A dependence of the width of the transition zone on the printing regime and heat input was also noted.

**Figure 7:**
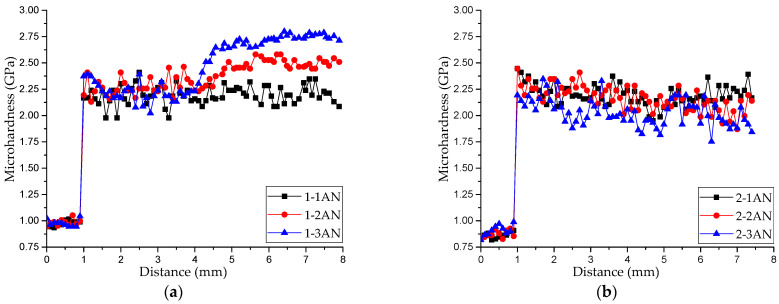
Distribution of the microhardness over the height of the annealed samples printed from Alloy 1 (**a**) and Alloy 2 (**b**).

Figure 7 presents the distributions of the microhardness along the height in the annealed samples. As can be seen from these plots, due to microstructural changes caused by annealing, the microhardness of the samples of series 2 and sample 1-1AN was within the same interval of values (2.0–2.4 GPa). This confirms the fact that annealing with quick cooling led to the transition of the structure to the martensitic phase, which can also be seen in Figure 5. Different properties of samples 1-2AN and 1-3AN can be explained by the fact that the transformation to the martensitic phase in them was restrained from the stable, ordered β_1_-phase.

### 3.3. Wear Analysis

**Figure 8:**
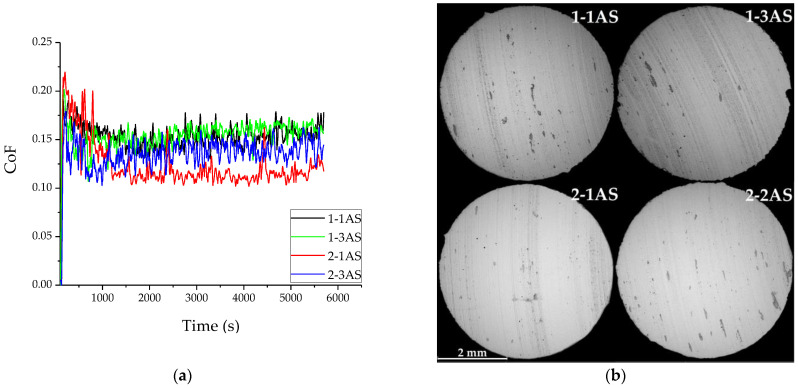
Coefficient of friction versus time for different samples (**a**) and SEM-BSE images of wear surfaces (**b**).

The results of pin-on-disc measurements are presented in Figure 8 and Table 4 for materials in different structural states. AS samples demonstrate a relatively low coefficient of friction in the range from 0.11 to 0.17. At the same time, the friction behavior of AS samples differs slightly among themselves. Because of the large amount of soft α-phase, sample 2-1AS had the lowest CoF, and sample 2-3AS had a CoF value between 2-1AS and samples 1-1AS and 1-3AS. However, all AS samples had a stable friction behavior with a short run-on stage and uniform CoF after process stabilization. The value of wear was also different for different printing conditions; however, in general, it was typical for aluminum bronze [30,31].

SEM analysis of friction surfaces (Figure 8b) showed a similar wear mechanism for all structure states. Oxides (the dark spots in Figure 8b) were formed during friction and were then crushed into smaller particles and smeared on the counterbody, forming a stable tribolayer. However, the difference in the structure and phase composition also led to uneven wear. For example, samples with a large amount of soft α-phase wore more quickly.

### 3.4. X-Ray Phase Analysis

**Figure 9:**
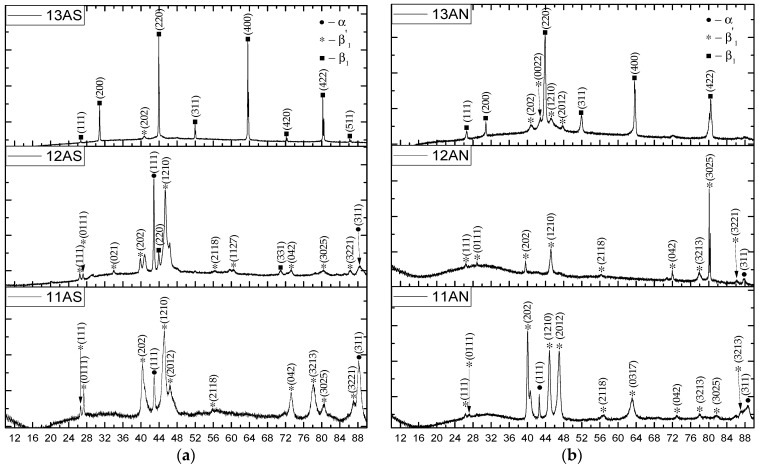
XRD diagrams of samples fabricated from Alloy 1: after printing (**a**); after annealing (**b**).

Cu–11Al–9Mn alloy was characterized by two types of phase composition depending on the printing regime. The samples with high heat input underwent eutectoid decomposition, as evidenced by XRD data (Figure 9). Depending on the printing regime, the ordered β_1_ phase either managed to complete the transition to α + β_1_′ (Figure 9c) or partially remained unchanged (Figure 9b). In the case of printing in the regime of the minimum permissible heat input (Figure 9a), the absence of Mn burnout and the high cooling rate made it possible to suppress decomposition, and the sample remained in the ordered β_1_ phase [8,16,26,32].

**Figure 10:**
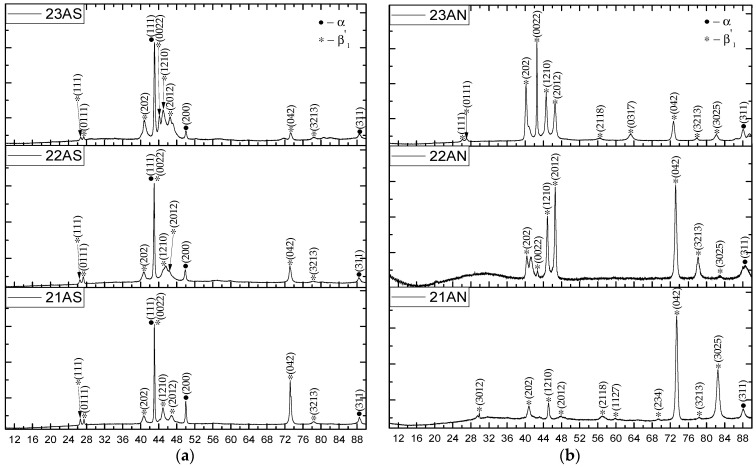
XRD diagrams of samples fabricated from Alloy 2: after printing (**a**), after annealing (**b**).

As can be seen from Figure 10, the phase composition of the printed Cu–11Al–4Mn layers strongly depended on the printing regime. Thus, the Cu–11Al–4Mn alloy samples were characterized by a structure with the presence of the following two phases: R18-type martensite and α-phase. The appearance of the α-phase was caused by the eutectoid decomposition, which is typical for bronzes [33,34]. As can be seen from the comparison of SEM images (Figure 4) and XRD diagrams (Figure 10), in the low-heat-input regime, the cooling of the sample was faster and, consequently, less α-phase had time to separate. Accordingly, as evidenced by the high degree of intensity of X-ray peaks, mainly R18 martensite was present in the sample. Samples printed at high heat input values contained much more α-phase since high beam energies during printing contributed to high sample heating. From the XRD diagrams of the samples subjected to annealing (Figure 10b,d,f), it can be seen that the heat treatment allowed them completely transform into the martensitic phase.

### 3.5. Discussion

The bulk samples from two alloys, Cu–11Al–4Mn and Cu–11Al–9Mn, were printed using the electron beam method at different heat input values. As an additional temperature treatment, some of the samples were annealed. The study showed a strict dependence of the structure-phase composition and mechanical characteristics on heat input during electron beam additive printing of the Cu–Al–Mn SMA alloys. It was established that the most optimal printing parameters corresponded to the value of heat input in the range from 0.24 kJ/mm to 0.72 kJ/mm, since they allowed obtaining a solid workpiece of the correct shape, without pores and melting defects, of controlled element-phase composition. Other authors also reported that alloys of aluminum bronze fabricated by different additive technologies with practically the same heat input had no pores or other defects [32,35,36]. The surface of the additively printed samples is usually not suitable for further practical use and must additionally postprocessed. For example, laser beam treatment can be used for the additional processing of the surfaces of the additively manufactured copper-based SMA [37].

The analysis of the microstructure by the SEM method showed a significant difference in the structure of samples printed with different heat input values. Thus, in the case of low heat input, the Cu–11Al–9Mn alloy sample remained in the ordered cubic β_1_′-phase, and large grains were formed. It is worth noting that the same structure had the samples of Cu–Al–N-–Mn bronze fabricated by selective laser melting in [32]. However, in the case of large values of heat input, the heat sink was insufficient, and the eutectoid decomposition had time to begin; the ordered β_1_′-phase decomposed into α and β.

The gradient zone formed in the samples also depended on the heat input: the less heat input, the narrower the gradient zone. Subsequent heat treatment eliminated the difference, and the width of the gradient zone for all printing regimes was smoothed. The structure-phase gradient also caused an uneven distribution of mechanical characteristics in the areas of the printed workpiece. Thus, the presence of a soft α-phase led to an uneven distribution of microhardness even in the upper layers, whereas the availability of solid β_1_ and β_1_′-phases contributed to an increase in mechanical characteristics and consequently wear resistance. It is known that an increase in the amount of aluminum increases microhardness, as well as wear resistance [30]. However, the friction process and CoF mainly depend not only on hardness, but also on microstructure. That is why annealed martensitic samples possessing hardness lower than β_1_ samples exhibited less wear. Intermetallic inclusions of solid phases also helped to significantly improve the characteristics of Cu–Al bronzes under dry friction [31].

Varying the heat input made it possible to control the structure, phase composition, and the structure-phase gradient. Thus, the variation of heat input when working with the raw material of Alloy 2 (Cu–11Al–4Mn) led to a significant change in the material. The formation zone of the β-phase narrowed due to changes in the chemical composition in the formed melt bath. In the process of further crystallization of the melt, the eutectoid decomposition of the β-phase into martensitic β_1_′ and α occurred. Depending on the printing regime, the melt had time to decompose (in the case of high heat input) or did not have time (in the case of low heat input). The above analysis confirms that the effect of heat input was much higher and provided more possibilities for the studied material than for Cu–Si–Mn alloys [38].

When printing from the raw material of Alloy 1 (Cu–11Al–9Mn) in the low-heat-input regime, eutectoid decomposition did not occur at all during crystallization, allowing the fabrication of a workpiece with a typical bronze structure consisting of grains elongated in the direction of the temperature gradient. Printing in the regime of high heat input also led to excessive burnout of Mn and the beginning of eutectoid decomposition. Cu–Al–Mn alloys containing about 10 at.% Mn and 15–23 at.% Al were also characterized by ferromagnetic behavior, which was caused by the formation of the ferromagnetic β_3_-phase of Cu_2_AlMn composition [39].

The phase composition affected wear performance since samples with a soft phase showed a lower CoF value. In general, the nature of the wear behavior and the CoF value dynamics of printed samples were comparable with the literature data for bronze alloys [19,30]. By and large, the friction mechanisms of alloys possessing a shape memory effect are much more complex, including the formation of a structured tribolayer (wear particles and oxides from both the sample and the counterbody) and deformation processes in the near-surface layer [40]. Factors such as texture and adhesion transfer also play an important role, which were discussed in detail in [41,42]. A detailed assessment of all the factors influencing the friction of additively printed SMA Cu–Al–Mn bronzes can be the subject of a more extensive study and should undoubtedly be considered in future.

## 4. Conclusions

On the basis of the results obtained from this study, the following conclusions can be made:

1. We showed that the method of combined wire and bar feeding is promising for electron-beam additive manufacturing of Cu–Al–Mn shape memory alloys. Due to the variation of print regimes and filaments, samples with different structures and phase compositions were obtained. Print regimes with small heat input proved to be the most effective because they allowed both avoiding defects and producing the required chemical composition of the material.
2. Varying the heat input in the electron-beam additive manufacturing was evaluated on the basis of the structure analysis of the fabricated samples. It was established that crystallization occurred directly into the β-phase, which allowed obtaining the material without additional heat treatment in the case of raw material 1 (Cu–11Al–9Mn). In the case of raw material 2 (Cu–11Al–4Mn), heat treatment was necessary in any case. Thus, samples in a state characterized by phase transformation, which caused the shape memory effect, were obtained by 3D printing with a combined technology of material supply and subsequent heat treatment.
3. Structure analysis and analysis of the microhardness distribution allowed us to describe the structure-phase gradient formed in the fabricated samples during EBAM. Tribological tests allowed us to evaluate the effect of the print regime on the wear performance of the fabricated samples. Thus, the materials with a higher content of soft phase had better sliding friction characteristics, while the materials containing hard phase possessed worse ones. Creating a directional gradient in layer-by-layer printing (by varying the printing parameters) allows controlling the mechanical behavior of the resulting sample in the most stress-prone areas.

## Figures and Tables

**Table 1:** Chemical composition of raw materials (wt.%).

Material	Cu	Al	Mn
Aluminum bronze (Cu–7Al)	93.5	6.3	0.2
Alloy 1 (Cu–11Al–9Mn)	79.2	11.2	9.6
Alloy 2 (Cu–11Al–4Mn)	85.1	11	3.9

**Table 2:** Selected parameters of the printing regimes.

Regime	Current *I*, mA	Voltage *U*, kV	Print Speed *V*, (mm/min)	Heat Input *E*, kJ/mm
0	40	30	50	1.44
1	40	30	100	0.72
2	40	30	200	0.36
3	40	30	300	0.24
4	40	30	400	0.18

**Table 3:** EDX analysis data (wt.%).

Sample	Cu	Al	Mn
1-1AS	83.0	10.6	6.4
1-2AS	80.9	10.9	8.2
1-3AS	79.6	11.1	9.3
2-1AS	87.1	10.8	2.1
2-2AS	86.1	10.8	3.1
2-3AS	85.5	10.8	3.7

**Table 4:** Wear rate of the fabricated samples in sliding friction.

Sample	Specific Wear Rate, mm^3^/(m∙N) × 10^−4^
1-1AS	1.10
1-3AS	1.05
2-1AS	1.32
2-3AS	1.22

## Data Availability

The data that support the findings of this study are available from the corresponding author upon reasonable request.
